# Effect of lignin fractions isolated from different biomass sources on cellulose oxidation by fungal lytic polysaccharide monooxygenases

**DOI:** 10.1186/s13068-018-1294-6

**Published:** 2018-10-28

**Authors:** Madhu Nair Muraleedharan, Dimitrios Zouraris, Antonis Karantonis, Evangelos Topakas, Mats Sandgren, Ulrika Rova, Paul Christakopoulos, Anthi Karnaouri

**Affiliations:** 1 Biochemical Process Engineering, Chemical Engineering, Department of Civil, Environmental and Natural Resources Engineering, Luleå University of Technology, Luleå, Sweden; 2 Laboratory of Physical Chemistry and Applied Electrochemistry, School of Chemical Engineering, National Technical University of Athens, Athens, Greece; 3 Biotechnology Laboratory, Department of Synthesis and Development of Industrial Processes, School of Chemical Engineering, National Technical University of Athens, Athens, Greece; 4 Department of Chemistry and Biotechnology, Swedish University of Agricultural Sciences, Uppsala, Sweden

**Keywords:** Lytic polysaccharide monooxygenases, Lignin structural properties, Electron donor, Cyclic voltammetry, Redox potential, Forest biomass, Pretreatment

## Abstract

**Background:**

Lytic polysaccharide monooxygenases (LPMOs) are copper-dependent enzymes that oxidatively cleave recalcitrant lignocellulose in the presence of oxygen or hydrogen peroxide as co-substrate and a reducing agent as electron donor. One of the possible systems that provide electrons to the LPMOs active site and promote the polysaccharide degradation involves the mediation of phenolic agents, such as lignin, low-molecular-weight lignin-derived compounds and other plant phenols. In the present work, the interaction of the bulk insoluble lignin fraction extracted from pretreated biomass with LPMOs and the ability to provide electrons to the active site of the enzymes is studied.

**Results:**

The catalytic efficiency of three LPMOs, namely *Mt*LPMO9 with C1/C4 regioselectivity, *Pc*LPMO9D which is a C1 active LPMO and *Nc*LPMO9C which is a C4 LPMO, was evaluated in the presence of different lignins. It was correlated with the physicochemical and structural properties of lignins, such as the molecular weight and the composition of aromatic and aliphatic hydroxyl groups. Moreover, the redox potential of lignins was determined with the use of large amplitude Fourier Transform alternating current cyclic voltammetry method and compared to the formal potential of the Cu (II) center in the active site of the LPMOs, providing more information about the lignin-LPMO interaction. The results demonstrated the existence of low-molecular weight lignin-derived compounds that are diffused in the reaction medium, which are able to reduce the enzyme active site and subsequently utilize additional electrons from the insoluble lignin fraction to promote the LPMO oxidative activity. Regarding the bulk lignin fractions, those isolated from the organosolv pretreated materials served as the best candidates in supplying electrons to the soluble compounds and, finally, to the enzymes. This difference, based on biomass pretreatment, was also demonstrated by the activity of LPMOs on natural substrates in the presence and absence of ascorbic acid as additional reducing agent.

**Conclusions:**

Lignins can support the action of LPMOs and serve indirectly as electron donors through low-molecular-weight soluble compounds. This ability depends on their physicochemical and structural properties and is related to the biomass source and pretreatment method.

**Electronic supplementary material:**

The online version of this article (10.1186/s13068-018-1294-6) contains supplementary material, which is available to authorized users.

## Background

The emerging environmental concern over fossil fuels depletion and exponential increase of the worldwide energy consumption have both triggered the search for alternative and sustainable energy resources [1]. Lignocellulosic biomass has recently gained much attention due to its abundance and great potential to replace not only petroleum-based fuels but also other chemicals in the current market [2]. In a biorefinery, lignocellulose-derived carbohydrates can be converted through enzymatic, chemo-enzymatic and biological processes into fermentable sugars that can be subsequently used as starting materials for the production of energy and value-added end products [3]. The currently available commercial carbohydrate-degrading enzyme cocktails targeted to efficiently degrade recalcitrant lignocellulose contain a wide range of cellulolytic and hemicellulolytic activities, as well as a novel class of oxidative enzymes, lytic polysaccharide monooxygenases (LPMOs) [4, 5]. LPMOs are one of the integral components of these cocktails due to their ability to boost the action of enzymes implied in the deconstruction of cellulose and hemicellulose [6, 7].

Lytic polysaccharide monooxygenases are copper active enzymes with a great gene diversity in the genome sequences of, among others, the lignocellulose degrading fungi [8]. Their mode of action differs from that of the hydrolytic enzymes due to their unique way of oxidative breakage of glycosidic bonds of the polymeric carbohydrates using molecular oxygen or hydrogen peroxide as co-substrate [9] in the presence of a reducing agent. Depending on whether they act on the C1 or C4 carbon atom of the glucose moiety, they have been classified as C1, C4 or mixed C1/C4 LPMOs [10]. C1 LPMO action generates a reducing end oxidized product, lactone, which is subsequently hydrolyzed to gluconic acid, while C4 LPMOs catalyze a non-reducing end oxidization to produce 4-ketoaldoses. The third type, C1/C4 LPMOs, exhibits a broader specificity and cleaves cellulose chain by oxidizing both C1 and C4 carbon atoms within the same polysaccharide chain. LPMOs have been shown to be able to utilize electrons from multiple sources including lignin [11], phenolic compounds [12–15], specific enzymes [13, 16, 17] or photosynthetic pigments [18]. The interaction of LPMOs with bulk insoluble lignin and soluble lignin derivatives is of pivotal importance for the enzyme system of wood degrading fungi; as in vivo, the saprophytic fungal lignocellulolytic systems possibly use lignin as reducing agent [19]. It has been also reported that the LPMO genes are highly up-regulated when the fungi act on lignocellulosic substrates [20]. Although some studies have been published [15, 16, 21], little is known about how properties of lignin, such as the molecular weight and the relative proportion of different units or hydroxyl groups, could affect the LPMO activity.

Different biomass pretreatment processes probably affect the oxidation efficiency of LPMOs and this is not only related to altered carbohydrate content and morphology [12]. Apart from the cellulose and hemicellulose content and structure that both greatly affect the biomass degradation process, the lignin properties are related to the ability of the substrate to provide electrons to LPMOs [14]. Lignin is a complex and recalcitrant biopolymer that consists of a network of three basic precursors, namely sinapyl, coniferyl, and *p*-coumaryl alcohols that form the lignin building blocks syringylpropane (S), guaiacylpropane (G), *p*-hydroxyphenol (H), respectively. The relative amount of S, G and H in lignin varies among the different types of wood; S and G units dominate in hardwood, while softwood lignin is rich in G units. Small amounts of H are present in both types of wood. The relative ratios of lignin aromatic units from agricultural residues differ among the species without any specific pattern observed. The content of S and G units has been correlated to the chemical reactivity of lignin, as β-*O*-4 bonds found in S lignin are more easily cleaved compared to that of G lignin [22]. That is reflected in the fact that hardwood delignification occurs in greater extent than in softwood under alkaline pretreatment conditions. Lignin methoxy content, the amount of aliphatic hydroxyl groups and the molecular weight of different lignins are among other factors that have been correlated with the lignin reactivity towards polymerization [23, 24]. Reactivity of lignin tends to increase with lower molecular mass lignins, increased ratios of ether linkages and aromatic to aliphatic hydroxyl groups, as well as lower amount of methoxy groups [23]. These factors are related to the type and the severity of the pretreatment followed and may affect lignin’s efficiency to serve as electron donor for the LPMOs.

In the present work, the activity of LPMOs on two types of forest biomass, hardwood (birch) and softwood (spruce), and wheat straw treated with different pretreatment methods were studied. Moreover, different lignin fractions isolated from these biomass samples were evaluated as potential electron donors for the LPMO-mediated degradation of phosphoric acid swollen cellulose (PASC). Three fungal enzymes were tested, namely *Mt*LPMO9 from *Thermothelomyces thermophila* (previously described as *Myceliophthora thermophila*) with C1/C4 regioselectivity [7], *Pc*LPMO9D from *Phanerochaete chrysosporium* which is a C1 active LPMO [25] and *Nc*LPMO9C from *Neurospora crassa* which is a C4 LPMO [26]. The lignin fractions were characterized in terms of their molecular size, the composition of aromatic and aliphatic hydroxyl groups and the reduction potential, which was compared to the reduction potential of the copper-containing active site of the LPMOs. For the calculation of the lignin and LPMO redox potential, large amplitude Fourier transform alternating current voltammetry (FTacV) was employed. This method offers advantages over the classical approach of protein film voltammetry which faces numerous difficulties arising from the low enzyme concentration and high capacitance currency [27]. With the FTacV method, the redox response can be isolated from all the factors that affect the signal, so it can be used even with low enzyme concentrations [28]. Moreover, FTacV allows for tracking the e^−^ transfer stages of an electrochemical reaction at various conditions of temperature and pH, which renders this method suitable for studying the activity of enzymes [29, 30]. This method has been used for the first time by Zouraris et al. to evaluate the redox potential of LPMOs by monitoring the direct e^−^ transfer between the enzyme and a glassy carbon electrode after LPMO immobilization in Nafion polyelectrolyte [31]. In this work, the study of the redox potential of LPMOs in concert with that of bulk lignin revealed whether the electron transfer was possible or not, in an attempt to shed light to the mechanism that LPMO employ to utilize lignin fractions as electron donors in the absence of an additional external mediator.

## Methods

### Pretreatment of forest biomass and isolation of different lignins

Wheat straw was pretreated hydrothermally in a microwave digestion equipment at 195 °C for 15 min as previously described [32]. Organosolv pretreatment of spruce and birch took place either in the presence or absence of acid as a catalyst. In the presence of H_2_SO_4_ 1% w/w_biomass_, biomass was treated for 60 min at 182 °C with 60% v/v ethanol [33]. In the absence of acid, spruce was treated with 52% v/v ethanol and birch with 60% ethanol and pretreatment took place at 200 °C for 30 min [34]. Steam explosion pretreatment was performed for 5 min, at 225 °C with H_2_SO_4_ 0.5% w/w_biomass_ for spruce and at 200 °C with H_2_SO_4_ 0.14% w/w_biomass_ for birch [35]. The cellulose, hemicellulose and lignin content of each material are listed in Table 1.

**Table 1:** Pretreatment conditions of different biomass and source of lignin fractions

	Hydrothermal	Steam explosion		Organosolv			
Acid catalyst	Yes	Yes		Yes		No	
Forest biomass	Wheat straw	Spruce	Birch	Spruce	Birch	Spruce	Birch
Pretreatment conditions	195 °C, 15 min	225 °C, 5 min, 0.5% w/w_biomass_ H_2_SO_4_	200 °C, 5 min, 0.14% w/w_biomass_ H_2_SO_4_	182 °C, 60 min, 60% v/v EtOH, 1% w/w_biomass_ H_2_SO_4_	182 °C, 60 min, 60% v/v EtOH, 1% w/w_biomass_ H_2_SO_4_	200 °C, 30 min, 52% v/v EtOH	200 °C, 30 min, 60% v/v EtOH
Compositional analysis of solid residues	50.2% w/w cellulose, 3.91% w/w hemicellulose, 25.5% w/w lignin	38.2% w/w cellulose, 0% w/w hemicellulose, 53.1% w/w lignin	57.2% w/w cellulose, 12.1% w/w hemicellulose, 27.1% w/w lignin	69.1% w/w cellulose, 1.15% w/w hemicellulose, 25.03% w/w lignin	60.73% w/w cellulose, 1.08% w/w hemicellulose, 15.69% w/w lignin	66% w/w cellulose, 6% w/w hemicellulose, 14.9% w/w lignin	67.1% w/w cellulose, 21.0% w/w hemicellulose, 7.1% w/w lignin
Lignins from solid residues	HT-A-WS	SE-S	SE-B	OS-A-S	OS-A-B	OS-S	OS-B
Lignins from liquid fraction				OS-A-S-LF	OS-A-B-LF	OS-S-LF	OS-B-LF

Lignin fractions used in this study were isolated: (i) from the liquid fraction, after organosolv pretreatment of the spruce and birch biomass (in the presence/absence of acid catalyst), (ii) from the residual solid fraction of pretreated biomass samples after cellulase/xylanase treatment and lignin isolation with dioxane–water (85:15 w/w) solution. The preparation of lignin samples is described below.

Lignin isolation and recovery from the pretreatment liquor was performed after water precipitation, as previously described [33, 34]. Briefly, the liquor was diluted with 1 L of cold deionized H_2_O in order to reach an ethanol content of less than 10% v/v and further reduce the solubility of lignin in the solution. The precipitated lignin was recovered with vacuum filtration, washed, freeze dried and stored thereafter. Lignin isolation from the solid biomass was performed as described previously [36]. Briefly, the residual solid fractions after pretreatment were treated with an enzyme mixture comprising cellulase and xylanase activities in order to achieve polysaccharide removal. Cellic^®^ CTec2 from Novozymes and Xyl6 xylanase from Dyadic were used at enzyme loadings of 20 FPU/g substrate and 0.25 mg/g substrate, respectively. Reactions took place for 24 h for 12 h at 50 °C and pH 5.0 (100 mM phosphate-citrate buffer) with an initial substrate concentration of 5% w/v dry matter. After polysaccharide removal, the enzymatically treated material was suspended in an acidified solution of dioxane–water (85:15 w/w) and subsequently refluxed for 4 h under nitrogen. The solution was then filtered, neutralized with NaHCO_3_ and added dropwise to acidified deionized water. The precipitated lignin was recovered after centrifugation and freeze drying. The pretreatment conditions and origin of lignins are detailed in Table 1.

### Characterization of isolated lignin properties

Isolated lignins were analyzed by gel permeation chromatography (GPC) and quantitative ^31^P NMR, as previously described [36], as well as Pyrolysis-gas chromatography–mass spectrometry (Pyr-GC/MS) to evaluate their properties (molecular weight, quantitative determination of various hydroxyl groups and monomeric composition).

For the GPC analysis, in order to increase the solubility of lignin in tetrahydrofuran (THF) [37], acetobromination took place after incubating 5 mg of lignin with 1 mL of glacial acetic acid/acetyl bromide (9:1 v/v) solution for 2 h. The solvent was evaporated and the residual solid was dissolved in THF and filtered, and 20 μL was injected to Waters Styragel HR-4E (Milford, MA, USA) column. The analysis was performed with at 40 °C with a mobile phase of THF at 0.6 mL/min. The number and weight-average molecular weights for each lignin were calculated using a polystyrene calibration curve (500–50,000 Da, Sigma-Aldrich) according to the previously published procedure [38].

For the ^31^P NMR analysis, 20 mg of each lignin was dissolved in 0.4 mL of pyridine/deuterated chloroform (1.6:1 v/v) and 0.1 mL of 2-chloro-4,4,5,5-tetramethyl-1,3,2-dioxaphospholane (95%, Sigma-Aldrich). Cholesterol was used as internal standard and chromium (III) acetylacetonate as the relaxation agent, as described elsewhere [36]. The mixture was incubated for 2 h at room temperature and analysis was performed based on previous literature reports [39].

For the Pyr-GC/MS analysis, the analytical pyrolysis system comprised a Shimadzu PY-3030S pyrolyzer coupled to a Shimadzu GCMS-QP2010 Ultra chromatograph with a quadrupole mass spectrometer detector (EI at 70 eV, ion source 240 °C). The column used was a Restek RTX-1701 (60 m × 0.25 mm, id 0.25 µm film thickness). The pyrolysis was carried out at 550 °C. A split ratio of 1:100 was used and the injection temperature was 280 °C. The gas chromatograph oven was held at 40 °C for 1 min and then ramped at 8 °C/min to 270 °C and then held at 40.25 min. Helium was used as carrier gas at a flow rate of 2.5 mL/min. Mass spectra were obtained for the molecular mass range *m/z* = 33–500. Samples were placed in a stainless-steel pyrolysis cup (PY1-EC50F, Frontier Laboratories Ltd.). At least two replicates per sample were carried out. The compounds were tentatively identified by comparing their mass spectra profiles to those in the NIST 2014 library. A minimum similarity of 80% was used for positive identification.

For the GC–MS analysis of soluble lignin extracts, three batches of approx. 10 mg of lignin each were extracted for 1 h at ambient temperature after an initial ultrasonication for 5 min with either 600 µL of toluene, or ethyl acetate or phosphate-citrate 100 mM buffer. In the latter case, the aqueous phase was acidified to pH 2 with diluted hydrochloric acid, extracted with 600 µL of ethyl acetate; the organic phase was separated and dried over magnesium sulphate. 500 μL aliquots of all final organic extracts were filtered by means of 0.45 μm syringe filter and treated with 75 µL of dry pyridine and 75 µL of *N*,*O*-bis(trimethylsilyl)trifluoroacetamide at room temperature approx. 30 min prior to analysis by gas chromatography coupled with mass spectrometry. Analysis was done using a Shimadzu GCMS QP2010 Ultra equipped with an AOi20 autosampler unit. An SLB^®^-5 ms Capillary GC Column (L × I.D. 30 m × 0.32 mm, df 0.50 μm) was used as stationary phase, ultrapure Helium as the mobile phase. The system was operated in ‘linear velocity mode’ with a starting pressure of 100 kPa, 280 °C injection temperature, and 200 °C interface temperature, running as temperature program: 50 °C start temperature for 1 min, 10 °C/min heating rate, 280 °C final temperature for 15 min). System control and analyses were realized using Shimadzu analysis software package Lab Solutions–GC MS solution Version 2.61. The various components were identified by comparison against the NIST11 library.

### Electrochemical measurements

Prior to the experiments, lignin to be tested was immobilized on the glassy carbon electrode. It was first dissolved in absolute ethanol at a concentration of 2 × 10^−3^ mg/mL. 1 μL of this solution was left to dry on the surface of the glassy carbon electrode for 2 min. This procedure was repeated two more times to increase the surface concentration of the lignin. After that 1 μL of Nafion was left to dry above the lignin on the electrode surface for few minutes. Concerning the immobilization of the enzymes the procedure is described elsewhere [31].

For the electrochemical study of the lignins and the LPMOs, a three-electrode cell was used consisting of a 1 mm disk glassy carbon electron as working electrode, a 1.6 mm diameter platinum-coated titanium rod as a counter electrode, and an Ag|AgCl, KCl sat. reference electrode (+ 0.197 V vs NHE at 25 °C). Cyclic voltammetry measurements were performed by a PAR 263A Potentiostat. For the FTacV experiments, an AFG 5101 Tektronix programmable arbitrary function generator was used together with the aforementioned potentiostat. Aqueous solutions of 100 mM tartrate buffer pH 5.0 were de-aerated by purging nitrogen gas for 5 min. Nitrogen gas was purged over the solution during the measurement in order to avoid interferences from the reduction of oxygen on the electrode surface. The temperature for the conducted experiments was 50 °C to ensure that the calculated values are determined under the same temperature as the other conducted experiments. Cyclic voltammetry was conducted at 5 mV/s for each lignin. Before recording the first measurement, about 10 cycles were performed at 100 mV/s, until a stable cyclic voltammogram was achieved. For the cyclic voltammetry of the reaction supernatants containing buffer and lignin-derived compounds (referred to as *supernatant 1*) and mixture of buffer, lignin and *Mt*LPMO9 (referred to as *supernatant 2*), the scan rates used were 30, 60, 100, 150 and 200 mV/s. The FTacV experiments were conducted for an amplitude of 280 mV and frequencies of 3, 6, 9 and 12 Hz for calculating the Eo of the enzymes active site and at 12.3 Hz and 110 mV for the isolated lignins and the supernatants. All the formal potentials estimated at 50 °C by taking into account the temperature coefficient − 1.01 mV/°C of the Ag|AgCl, KCl sat. reference electrode [40].

### Determination of LPMO activity in the presence of different lignin fractions

The activity of three different LPMOs (*Pc*LPMO9D, *Nc*LPMO9C and *Mt*LPMO9) on pretreated wheat straw, spruce and birch was evaluated in the presence or absence of ascorbic acid. Reaction conditions were: 3% w/v initial dry matter (DM) in 400 μL final reaction volume, enzyme loading 30 mg/g substrate (protein concentration determined by Lowry method) [41], 100 mM phosphate-citrate (in case of *Pc*LPMO9D and *Mt*LPMO9) or sodium acetate (in case of *Nc*LPMO9C) buffer, incubation at 50 °C, for 24 h, under agitation (1100 rpm). Ascorbic acid was added at a final concentration of 1 mM. Samples were boiled for 5 min to deactivate the enzyme, cooled down and filtered and soluble products were analyzed by HPAEC-PAD. Blank reactions containing lignocellulosic substrate and buffer, substrate and LPMO (no reducing agent), or substrate and ascorbic acid were also analyzed. All blank reactions showed small peaks in the C1–C3 area of neutral sugars. Peak assignment of oxidized sugars was done according to previous data [42].

The ability of the LPMOs to use the lignins isolated from forest materials as electron donors and release oxidized sugars from PASC was evaluated and compared with the enzymatic activity in the presence of ascorbic acid. PASC was prepared from Avicel as previously described [43]. Reaction conditions and sample analysis were the same as described above for the natural substrates, with the difference that the reactions were carried out using 2% w/v initial DM and an enzyme loading of 25 mg/g substrate. Ascorbic acid was added at a final concentration of 1 mM, while lignin loading was 10 mg/mL. Blank reactions containing PASC and LPMO (no reducing agent) were also analyzed.

## Results and discussion

### Properties of isolated lignins

**Fig. 1:**
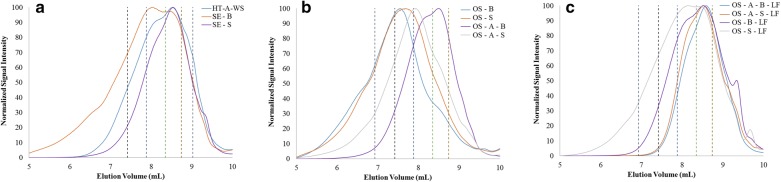
GPC chromatograms for (**a**) HT-A-WS, SE-B, SE-S lignins, (**b**) OS-B, OS-S, OS-A-B, OS-A-S lignins and (**c**) OS-A-B-LF, OS-A-S-LF, OS-S-LF, OS-B-LF lignins, with dotted lines indicating the elution volume for some of the polystyrene standards (500, 1000, 2000 and 4000 Da)

The weight-average molecular weight (*M*_w_), number-average molecular weight (*M*_n_) and dispersity index (PDI) of the lignin fractions, as determined using GPC analysis, are summarized in Table 2, while the GPC chromatograms are depicted in Fig. 1. All lignins isolated from the liquid fractions were of lower molecular weight, compared to the ones extracted from the solid fractions. Lignins from the liquid fractions of acid-catalyzed organosolv pretreated spruce and birch (OS-A-S-LF and OS-A-B-LF) showed the lowest molecular weights and more uniform mass distribution with a PDI value lower than other lignins. Among the lignins from spruce solid fractions, that from steam exploded material (SE-S) showed the lowest molecular weight, while in case of birch that was observed in organosolv-treated biomass in the presence of acid catalyst (OS-A-B). The lignin isolated from SE-S, OS-S and OS-S-LF exhibited lower molecular weight than those isolated from birch. The opposite was observed when OS pretreatment was combined with an acid catalyst; OS-A-S exhibits lower molecular weight than OS-A-B. There was also a slight reduction in molecular weight of both spruce and birch lignins from organosolv liquid fraction when the acid catalyst was added. This result is also depicted in the data from the residual solid fractions. As expected, in the organosolv processes, the use of acid treatment generates much lower molecular weight lignins, compared to that without acid, both in solid and liquid fractions. The presence of a catalyst promotes the cleavage of lignin aryl-ether bonds, releasing lignin fractions with lower molecular weight [44]. The effect of acid treatment was more obvious on birch lignins (5.6 times lower molecular weight in solid fractions and 4.5 times lower in liquid fractions), while in spruce it was 1.5 and 1.7, respectively.

**Table 2:** Weight-average molecular weight (*M*_w_), number-average molecular weight (*M*_n_) and dispersity index (PDI) of the lignin fractions samples from various pretreated biomass

	*M*_n_	*M*_w_	PDI
HT-A-WS	1262	3819	3.03
SE-B	1798	13,447	7.48
SE-S	1138	2886	2.54
OS-B	3041	19,714	6.48
OS-S	2794	16,543	5.92
OS-A-B	1364	3488	2.56
OS-A-S	1987	9595	4.83
OS-B-LF	1442	8041	5.58
OS-S-LF	1017	2832	2.79
OS-A-B-LF	983	1780	1.81
OS-A-S-LF	980	1890	1.93

Quantitative assessment of lignin units was done by ^31^P NMR (Table 3) and Pyr GC/MS (Table 4) analysis, with the aim to determine both the total aliphatic and aromatic hydroxyl groups and the individual S, G and H content, respectively. The quantitative results are presented as mmol/g lignin. The free phenolic/aliphatic hydroxyl groups in the lignins were phosphorylated prior to the analysis and were later identified by ^31^P NMR. The free aromatic –OH groups contribute to the reactivity/reducing potential of the lignins. Addition of acid catalyst in the organosolv treatment greatly changed the aromatic/aliphatic –OH groups ratio. A decrease in aliphatic –OH combined with a simultaneous increase in aromatic –OH content was observed when acid was added in organosolv treatment and this was more evident in case of birch lignins. A higher amount of aromatic –OH groups indicates higher reactivity of lignin, while a lower amount of aliphatic –OH groups denotes a much intense pretreatment [45]. The increase in phenolic –OH content is a result of β-*O*-4 linkages cleavage and leads to a greater fragmentation of the lignin structure [46], as verified by the molecular weight reduction in the above results. It was also observed that the lignins from the liquid fractions of acid-catalyzed organosolv pretreatment (OS-A-S-LF and OS-A-B-LF) had the highest amount of free –OH groups (Table 4), which would make them the most reactive among other lignin fractions. In Pyr-GC/MS analysis, lignin samples were decomposed due to the higher temperature and inert atmosphere to produce smaller molecules that were separated by gas chromatography and detected in a mass spectrometer. Pyr-GC/MS data provided the total composition of S, G, and H units which comprise both the reactive (free phenolic groups) and the non-reactive (phenolic groups bonded to others) units. The total S, G, H values showed important information about the source of lignins. Birch as a hardwood had both S and G units, while spruce being softwood had G as the main building unit. The high amount of S units in lignin isolated from birch indicated a more reactive material as the β-*Ο*-ether of syringyl lignin is cleaved much easier than that of guaiacyl lignin [47].

**Table 3:** Estimated aliphatic and aromatic groups content (mmol/g) of different lignins as evaluated from ^31^P NMR

	Aliphatic OH	Aromatic OH	Arom-/aliphatic OH
HT-A-WS	1.08	1.82	1.68
SE-B	3.45	1.46	0.42
SE-S	1.19	2.76	2.31
OS-B	7.26	0.97	0.13
OS-S	1.95	1.78	0.91
OS-A-B	1.02	1.58	1.76
OS-A-S	0.95	2.28	2.40
OS-B-LF	3.22	1.69	0.52
OS-S-LF	2.97	2.45	0.83
OS-A-B-LF	0.67	3.25	4.84
OS-A-S-LF	1.57	3.15	2.01

**Table 4:** Content of estimated total aromatic monolignols (S, G, H) of different lignins as determined using Pyr GC/MS

	S	G	H	G/S	H/S
HT-A-WS	26	57	18	2.19	0.69
SE-B	64	32	4	0.50	0.06
SE-S	3	88	9	29.30	3.00
OS-B	68	29	3	0.43	0.04
OS-S	2	90	9	45.00	4.50
OS-A-B	51	37	12	0.73	0.24
OS-A-S	2	87	11	43.50	5.50
OS-B-LF	61	36	3	0.59	0.06
OS-S-LF	1	91	7	69.39	5.71
OS-A-B-LF	58	36	6	0.62	0.11
OS-A-S-LF	2	89	9	54.37	5.31

### Estimation of reduction potential of lignin fractions and LPMOs

Lignin contains 15–30 free phenolic OH groups per 100 of C_9_ units in softwoods and 10–15 groups in hardwoods [48]. When oxidation of this phenolic hydroxyl group occurs, the first step is the formation of a phenoxy radical I followed by de-methoxylation and formation of an *o*-quinone II. Phenoxy radicals can also recombine and form new C–C and O–O bonds leading to cross-linking of the lignin structure [49]. The presence of a redox system assigned to lignin-derived quinone/hydroquinone couple can be tracked through cyclic voltammetry, allowing for the estimation of the lignin redox potential. Concerning LPMOs, the only estimation of their redox potentials in the literature has been done indirectly, by measuring the products of the action of an LPMO enzyme or through the use of mediators [17, 50–52].

**Fig. 2:**
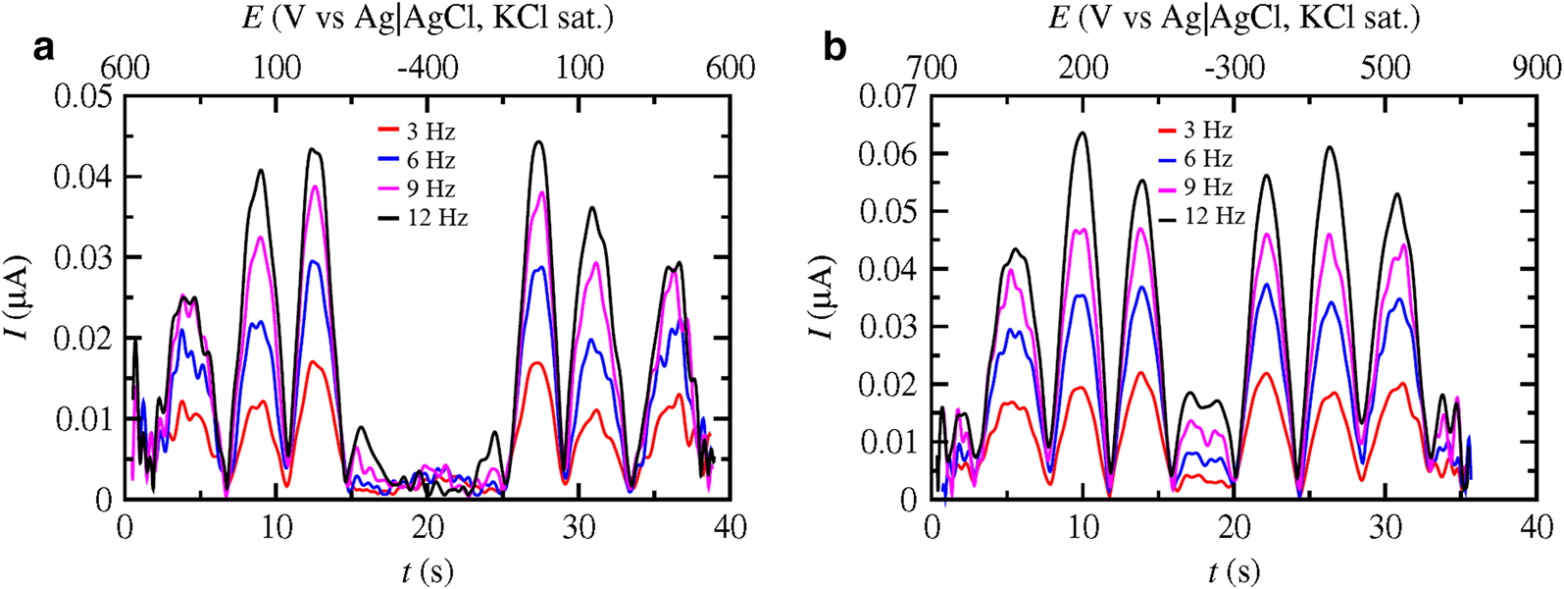
FTacV 5th Harmonic of **a** *Pc*LPMO9D and **b** *Nc*LPMO9C immobilized on a glassy carbon electrode with the use of Nafion for a *v *= 50 mV/s, *A* = 280 mV and different frequencies in de-aerated 100 mM tartrate buffer pH 5.0 at 50 °C

The formal potentials of *Pc*LPMO9D and *Nc*LPMO9C were determined with FTacV. As can be seen in Fig. 2 the 5th harmonic from the FTacV analysis of the two LPMOs is presented at different frequencies. The formal potentials are taken as an average of the values of the middle peaks for each frequency. The respective potentials were estimated at 154.2 ± 2.1 and 214.8 ± 2.1 mV at 50 °C and pH 5.0, respectively. The formal potential of *Mt*LPMO9 has been estimated to be equal to 119.6 ± 7.1 mV under the same conditions in a previous work [31].

The cyclic voltammograms of the immobilized lignins can be found in Additional file 1: Figure S1 for a scan rate of 5 mV/s. For an electrochemically reversible reaction of an immobilized species, the separation of the oxidation and the reduction peaks should be close to zero, in order to estimate the formal potential of the species. In the case of the immobilized lignins, it can be observed that even at a relatively low scan rate of 5 mV/s the peak separation is well above 100 mV, indicating a quasi-reversible reaction, thus not securing a safe estimation of the formal potential. What can be assumed safely is that the formal potential lies somewhere between the values of the oxidation and the reduction peaks for each lignin, which can be found in Table 5.

**Table 5:** Calculated values for the oxidation *E*_ox_, reduction *E*_red_ and formal potential *E*^o^’ estimated with cyclic voltammetry and FTacV for lignins immobilized on a glassy carbon electrode with the use of Nafion in 100 mM tartrate buffer pH 5.0 at 50 °C

	Cyclic Voltammetry		FTacV		
	*E*_ox_	*E*_red_	*E*_ox_	*E*_red_	*E*_o_’
HT-A-WS	387	281	322.3 ± 11.2	320.2 ± 17.9	346.5 ± 21.1
SE-B	361	241	325.7 ± 7.8	306.9 ± 16.5	316.3 ± 18.2
SE-S	359	241	357.1 ± 11.7	339.3 ± 7.2	348.2 ± 13.8
OS-B	358	274	283.3 ± 3.4	337.1 ± 23.3	310.2 ± 23.6
OS-S	368	258	363.7 ± 11.1	352.7 ± 19.8	358.2 ± 22.7
OS-A-B	367	272	310.2 ± 24.1	303.9 ± 9	307.1 ± 33.1
OS-A-S	361	271	347.5 ± 21.5	315.4 ± 27.6	331.4 ± 31.9
OS-B-LF	355	218	269.9 ± 11.9	229.9 ± 19.1	249.9 ± 23.8
OS-S-LF	356	252	349.6 ± 12.5	309.1 ± 32.1	354.7 ± 34.5
OS-A-B-LF	358	223	264.6 ± 34.2	197.0 ± 18.6	230.8 ± 47.9
OS-A-S-LF	353	190	278.4 ± 20.0	209.8 ± 22.7	256.7 ± 30.2

Another approach to estimate the formal potentials of the immobilized lignins was with the use of the FTacV method [28]. In (Additional file 1: Figure S2), the set of the first six Harmonics is presented for HT-A-WS lignin as an indicative example of the voltammograms resulting from this method. The formal potential was calculated from the average of the oxidation and reduction main peak for the odd harmonics and the mid minimum for the even harmonics. The 3rd through 6th harmonics were used, where the non-faradaic currents have been eliminated and the formal potential is calculated as the average of the calculated potentials for the aforementioned harmonics. The reduction, oxidation as well as formal potentials estimated for each lignin along with their standard deviations are presented in Table 5. Firstly, it is observed that the formal potentials estimated with FTacV are within the limits indicated by the cyclic voltammograms. It was then observed that HT-A-WS and all lignin samples originating from spruce exhibited higher potentials than the ones originating from birch. Moreover, the lignin isolated from pretreated materials upon the addition of acid catalyst (OS-A-S-LF and OS-A-B-LF) appeared to have lower potentials. The redox potential values found for both the immobilized lignins [49, 53–55] and the LPMOs [17, 50–52] are in accordance with those that have been estimated in the literature.

### Evaluation of LPMOs activity on pretreated forest and agricultural residues

**Fig. 3:**
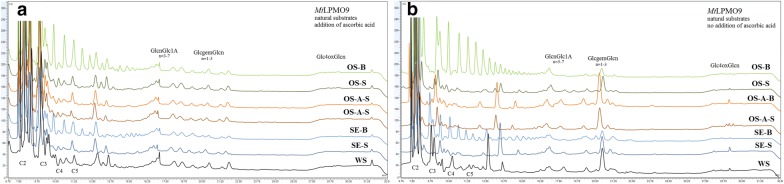
The products of *Mt*LPMO9 action on different substrates with (**a**) or without (**b**) the addition of ascorbic acid as an additional external electron donor. Products at 5–13 min correspond to neutral sugars, 13–19 min to C1 oxidized sugars, 19–25 min to C4 oxidized sugars and 25–30 min to mixed C1/C4 oxidized sugars. Peaks at 12.3, 21.8 and 31.6 min are assigned to ascorbic acid

**Fig. 4:**
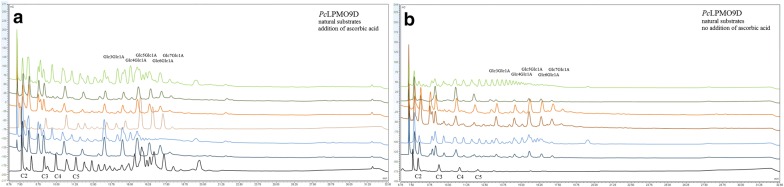
The products of *Pc*LPMO9D action on different substrates with (**a**) or without (**b**) the addition of ascorbic acid as an additional external electron donor. Products at 5–13 min correspond to neutral sugars and 13–19 min to C1 oxidized sugars

**Fig. 5:**
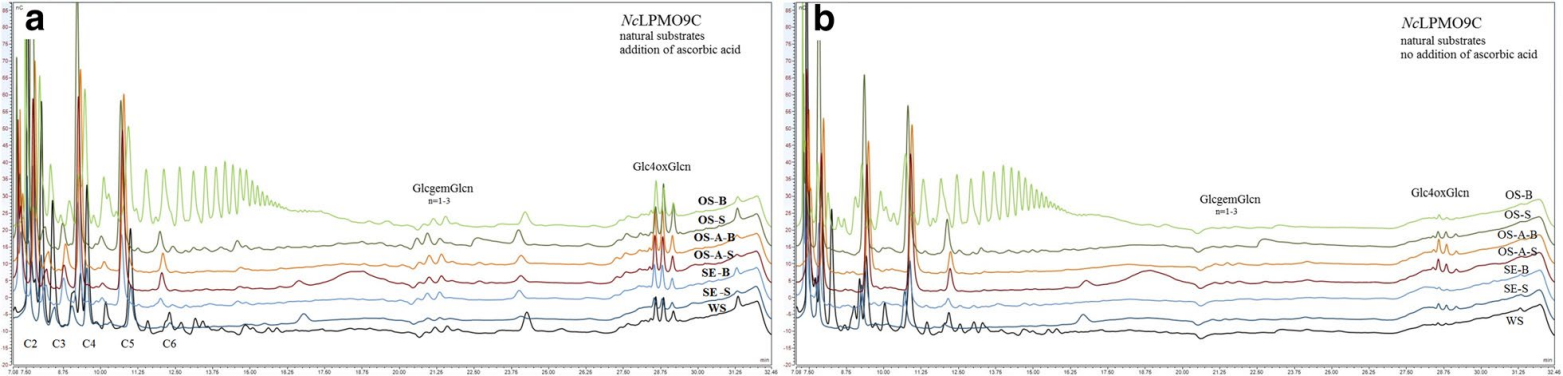
The products of *Nc*LPMO9C action on different substrates with (**a**) or without (**b**) the addition of ascorbic acid as an additional external electron donor. Products at 5–13 min correspond to neutral sugars, 19–25 min to C4 oxidized sugars and 25–30 min to mixed C1/C4 oxidized sugars

The activity of *Mt*LPMO9, *Pc*LPMO9D, *Nc*LPMO9C on hydrothermally pretreated wheat straw, steam exploded and organosolv pretreated birch and spruce was evaluated with or without the addition of ascorbic acid as external donor. *Mt*LPMO9 showed the maximum release of sugars in the absence of ascorbic acid (Fig. 3), while *Pc*LPMO9D (Fig. 4) and *Nc*LPMO (Fig. 5) preferred ascorbic acid to the lignin that was present in the substrate. The activity of all three LPMOs was generally higher on spruce compared to birch and on steam exploded and organosolv-pretreated materials upon the addition of acid catalyst. The activity of LPMOs on the natural substrates is correlated with the pretreatment method and the lignin characteristics, as described above. Among the solid fractions after pretreatment, the spruce fraction had the lignin with the lowest molecular weight and highest aromatic content after steam explosion pretreatment, and for birch it was from organosolv treatment in the presence of acid catalyst, justifying the higher sugar yield from these substrates. In the organosolv treatments, the presence of acid catalyst greatly reduced the molecular weight of lignins. It was also observed that acid treatment increased the amount of aromatic and reduced the aliphatic OH groups, producing a more reactive lignin fraction that could trigger the LPMO activity. It was previously reported that the amount of aliphatic –OH groups decreases with increase in the intensity of pretreatment [31]. During acid-catalyzed treatment, aliphatic bonds break and other compounds such as HBF (hydroxybenzofuran) are formed via dehydro-cyclization of aliphatic –OH group, which could be the reason of the reduction in their number [56]. Different linkages between the lignin units and the various functional groups render this compound complex and with a unique structure, while primarily affecting its overall reactivity.

The selection of pretreatment method is crucial for the subsequent depolymerization and overall valorization of lignocellulosic biomass, as it affects the lignin properties by promoting chemical and structural modifications. It has been assumed that organosolv lignins and organosolv-pretreated solid fractions are not able to provide electrons to LPMOs, not only because the main part of them is cleaved and dissolved in the liquid fraction, leaving a lignin-free solid pulp, but also because condensation reactions leading to lignin repolymerization occur [57], which is also depicted in our case in the absence of catalyst and verified by the GPC analysis above. Lignin repolymerization leads to production of lignins with higher molecular weight and chemical characteristics that affect their ability to provide electrons [14].

### Evaluation of different lignin fractions as external electron donors to LPMOs

**Fig. 6:**
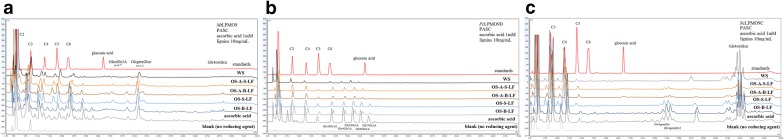
The products of **a** *Mt*LPMO9, **b** *Pc*LPMO9D and **c** *Nc*LPMO9C action on PASC in the presence of lignin isolated from the pretreated materials. Ascorbic acid was added to a final concentration of 1 mM and lignin to 10 mg/mL. Products at 5–13 min correspond to neutral sugars, 13–19 min to C1 oxidized sugars, 19–25 min to C4 oxidized sugars and 25–30 min to mixed C1/C4 oxidized sugars

The ability of *Mt*LPMO9, *Pc*LPMO9D, *Nc*LPMO9C to use lignin fractions isolated from agricultural and forest materials as electron donors and release oxidized sugars from regenerated cellulose was evaluated and compared with the activity in the presence of ascorbic acid. All three enzymes showed the maximum release of sugars in the presence of ascorbic acid (Fig. 6) and were able to use all lignin types as reducing agents for the deconstruction of cellulose resulting in the release of oxidized sugars. Isolated lignin boosts *Mt*LPMO9 more than *Pc*LPMO9D and *Nc*LPMO9C compared to the reactions in the presence of ascorbic acid. Lignin isolated from the liquid fractions of acid-catalyzed organosolv pretreatment (OS-A-S-LF and OS-A-B-LF) served as better electron donors for the *Mt*LPMO9, *Pc*LPMO9D and *Nc*LPMO9C compared to other fractions (Additional file 1: Figure S3, S4, S5). The results were in accordance with those observed for the real substrates, verifying that LPMO performance was greater in the presence of SE-S and OS-S compared to SE-B and OS-B, respectively, while they preferred OS-A-B, OS-B-LF and OS-B-A-LF rather than the corresponding lignin fractions from spruce. The lignin structure holds a key role as it determines how easy it is to provide electrons to the low-molecular weight phenolic compounds and, finally, to LPMOs. In general, the lignins from liquid fractions exhibited lower molecular weights and higher aromatic content than the ones from solid fractions. Three of the lignins that showed the highest activity were extracted from the liquid fraction of organosolv pretreatment and among them the best ones were from acid treatment. For spruce, pretreatment by steam explosion resulted in residual lignocellulose with highest aromatic –OH and low-molecular weight compared to the organosolv method. This shows the importance of pretreatment of biomass targeting LPMOs and LPMO containing cellulase cocktails. Apart from redox potential values and other characteristics, such as S-unit content, the concentration is also a key factor. As the amount of lignin added in these experiments is based on the total weight (mg/mL of reaction), lignins with lower molecular weight possess more reactive sites as they have higher amount of free phenolic groups, which enables them to interact and provide electrons more easily. The composition of the functional groups of lignin and their physical distribution is different, which also affects their electron-providing capacity.

### Ability of LPMOs to use low-molecular weight lignin-derived soluble compounds and further characterization of their properties

From the electrochemical results described above, it can be concluded that the values of lignin formal potentials are higher than the ones of *Mt*LPMO9, *Pc*LPMO9D and *Nc*LPMO9C, indicating that the redox reaction between these species is not thermodynamically feasible. It should be highlighted that the formal potentials estimated for the lignins correspond to the charge transfer reaction of the lignins on the electrode surface. The lack of feasibility of the reaction between the lignins and the LPMOs based on the calculated formal potentials does not exclude the possibility of another oxidation path of the lignins that is not detectable electrochemically. Additionally, it has been proposed that the exchange of electrons between the lignin and the LPMOs is not direct but occurs through the use of lower molecular weight compounds [15]. To further verify that the LPMOs are able to utilize the phenolic compounds that are released in the reaction medium, another set of experiments was conducted. HT-A-WS lignin was incubated either alone or in the presence of *Mt*LPMO9 in citrate–phosphate buffer 100 mM pH 5.0 for 24 h at 50 °C under agitation and the reaction mixtures were then centrifuged. The supernatants were collected and analyzed with cyclic voltammetry, FTacV and GC–MS, while the precipitates were washed two times with buffer and analyzed with GPC and 31P NMR. All fractions were used with PASC and *Mt*LPMO9, in order to evaluate their ability to provide electrons to the enzyme active site.

**Fig. 7:**
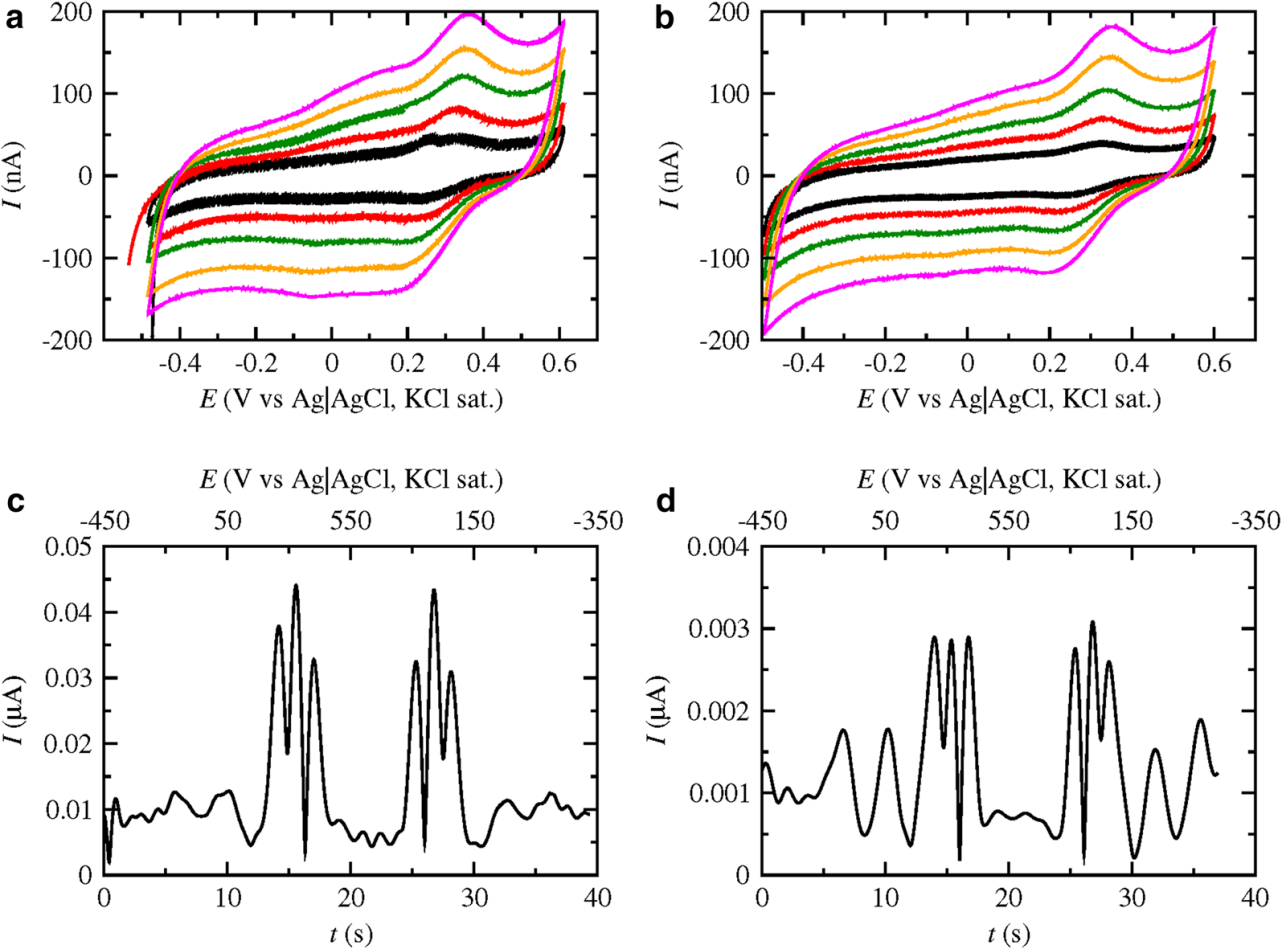
Cyclic voltammograms of supernatants after **a** lignin and **b** lignin in the presence of *Mt*LPMO9 incubation in 100 mM phosphate-citrate buffer pH 5.0 for 24 h at 50 °C, at 30 (*black*), 60 (*red*), 100 (*green*), 150 (*orange*) and 200 (*mangeta*) mV/s in de-aerated 100 mM tartrate buffer pH 5.0 at 50 °C. FTacV 5th Harmonic of **c** HT-AWS and **d** supernatant 1 immobilized on a glassy carbon electrode with the use of Nafion for a *v* = 50 mV/s, *A* = 110 mV, *f *= 12.3 Hz in de-aerated tartrate buffer 100 mM pH 5.0 at 50 °C

The supernatant after lignin incubation without the addition of the enzyme (*supernatant 1*) was immobilized on the glassy carbon electrode. Both cyclic voltammetry and FTacV were performed under the same conditions as for the immobilized lignins. In Fig. 7a, the cyclic voltammetry experiments are depicted at different scan rates and it is evident that a second redox peak couple appears around 20 mV apart from the one around 300 mV, that is the one corresponding to the redox couple observed at the voltammetric experiments of HT-A-WS immobilized on the electrode surface. Comparing the 5th harmonic of HT-A-WS (Fig. 7c) and *supernatant 1* (Fig. 7d), a second set of peaks is arising at around 20 mV in the supernatant, reinforcing the indication of the cyclic voltammogram that a second redox peak appears. The above results indicate that some electroactive compounds possibly derived from the bulk lignin structure can be released in the solution during the incubation and the electron transfer is possible through them. The potential of the second redox couple is more negative than the one of the LPMOs indicating that their oxidation by the LPMOs is feasible. The supernatant of lignin incubated with *Mt*LPMO9 (*supernatant 2*) is also analyzed with cyclic voltammetry under the same conditions (Fig. 7b), showing that the second redox couple is less evident, thus indicating that the couple has reacted and been consumed (oxidized). The results indicate that LPMOs are able to oxidize the lignin-derived electroactive compounds that are released in the reaction in a thermodynamically feasible process, indicating that these compounds act as the initial reducing agents that trigger the action of the enzymes.

31P NMR and GPC analysis of the precipitants did not show any significant detectable difference of the HT-A-WS lignin prior and after incubation with buffer and MtLPMO9/buffer (Additional file 1: Figure S6 and Table S1). Although 31P NMR showed a slight increase in the amount of aliphatic OH indicating that some aromatic compounds that are found on the bulk lignin surface can diffuse into the liquid fraction and thus removed from the insoluble lignin structure, but the difference is very low to extract a safe conclusion. GPC also failed to show any significant difference. GC–MS analysis of the supernatant 1 was performed after extraction of the reaction supernatant with ethyl acetate. Results (Additional file 1: Table S2) showed the presence of a benzaldehyde analog (syringaldehyde) that is possible one of the compounds that serve as mediators to shuttle electrons from lignin to LPMOs. Throughout the literature, phenolic compounds such as vanillic acid, *p*-coumaric acid, and ferulic acid have been identified as possible electron donors to LPMOs and have been studied extensively [13, 15, 16]. In this study, the existence of low-molecular weight mediators that shuttle electrons between the bulk insoluble lignin and the active site of the LPMOs is verified. These soluble compounds hold a key role in the initial reduction step, and, in case of insoluble reductants, it could be that LPMOs strictly require additional external intermediates for their action [12].

**Fig. 8:**
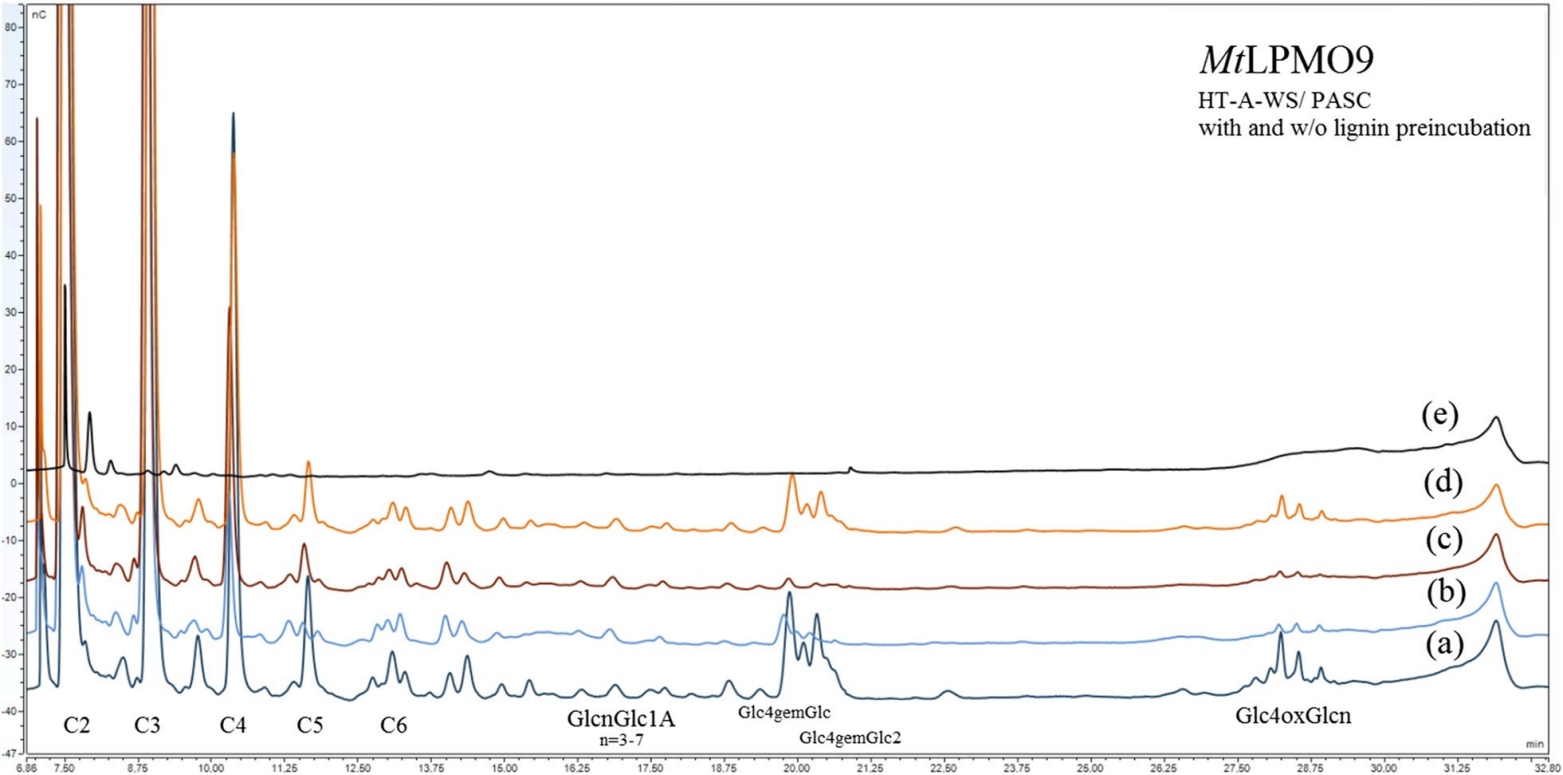
The products of *Mt*LPMO9 action on PASC in the presence of HT-A-WS lignin after incorporating a lignin pre-incubation step in buffer, centrifuge the reaction mixture and using either the supernatant or the precipitate as a reducing agent for the enzyme activity. **a** *Mt*LPMO9 and lignin added simultaneously on PASC and incubated for 24 h, **b** lignin was pre-incubated in buffer for 24 h, then the supernatant was added to PASC together with *Mt*LPMO9 and incubated for another 24 h, **c** lignin was pre-incubated in buffer for 24 h, then the precipitant was added to PASC together with *Mt*LPMO9 and incubated for another 24 h, **d** PASC was pre-incubated with lignin for 24 h, then *Mt*LPMO9 was added and incubated for another 24 h, **e** lignin and PASC were incubated for 24 h

Both supernatants and precipitates were tested for their ability to promote the activity of *Mt*LPMO9 on PASC acting as external electron donors. It was shown that the supernatant could act as a reducing agent (Fig. 8) and support the release of oxidized sugars from PASC. The sugars yield was much lower when lignin was used, which is attributed to the consumption (oxidation) of the free phenolic compounds that terminated the reaction. Interestingly, when the lignin precipitate was used, only traces of oxidized sugars are detected. One additional reaction where PASC and HT-A-WS lignin were pre-incubated together for 24 h, at 50 °C also led to lower yield of oxidized sugars, showing that possible changes may have occurred either in the cellulosic substrate or the lignin fraction. These results combined with the relative redox potential values of the enzymes and the lignin fractions justify the biochemical experiments, leading to the assumption that LPMOs do not use directly the bulk lignin as an electron donor, but the small molecules that are possibly released in the buffer instead.

## Conclusions

The aim of the present work was to obtain a better understanding of the interaction between the lignin remaining in the biomass after pretreatment and the oxidative reactions of LPMO enzymes. Many studies have discussed about different possible electron sources for LPMOs, such as various enzymes and light pigments. Recently, it was shown that several low-molecular weight lignin-derived compounds formed by the action of lignin-active enzymes that are co-expressed along with LPMOs can act as electron donors for LPMO action by a process of shuttling between insoluble bulk lignin and LPMO. In this current study, we verify that LPMO indeed can access bulk lignins as electron source for their action by initially reducing the phenolic compounds that are released from lignin in the reaction medium. *Mt*LPMO9, *Pc*LPMO9D and *Nc*LPMO9C are able to utilize electrons from the phenolic compounds that are released from the insoluble bulk lignin fractions of varying molecular weights and compositional properties that have been isolated from different biomass sources, in order to oxidatively break cellulose. Among the lignins, the ones from the liquid fractions of acid-catalyzed organosolv pretreatment of spruce and birch (OS-A-S-LF and OS-A-B-LF) were the most successful in supporting LPMO action. Further evaluation of the properties of the isolated lignins using a series of analytical methods revealed more information why these two lignin fractions served as the best ones. It was found that these fractions had the lowest molecular weights and a more uniform mass distribution, as well as the highest amount of aromatic OH. The efficiency of lignins to successfully transfer electrons to low-molecular weight phenolic compounds and, finally, to LPMOs is not dependent on one factor, but on different parameters, such as the amount and the positioning of aromatic OH, the relative amount of S, G, H units, the molecular size of lignin, the polydispersity index and the reduction potential value. However, in a general outlook it could be concluded that a low-molecular weight and high aromatic content are key factors that are related to the reduction potential of lignin fractions and their ability to provide electrons.

## Additional file

**Additional file 1: Figure S1.** Cyclic voltammograms of (**A**) HT-A-WS, (**B**) SE-S, (**C**) SE-B, (**D**) OS-S, (**E**) OS-B, (**F**) OS-A-S, (**G**) OS-A-B, (**H**) OS-S-LF, (**I**) OS-B-LF, (**J**) OS-A-S-LF and (**K**) OS-A-B-LF immobilized on a glassy carbon electrode with the use of Nafion in 100 mM tartrate buffer pH 5.0, at 50 ^o^C between -200 and 600 mV vs Ag/AgCl for a scan rate of 5 mV/s. **Figure S2.** FTacV 1^st^ to 6^th^ Harmonic of HT-A-WS lignin immobilized on a glassy carbon electrode with the use of Nafion for a *v =* 50 mV/s, A = 110 mV*, f* = 12.3 Hz in de-aerated 100 mM tartrate buffer pH 5.0 at 50^ο^C. **Figure S3.** The products of *Mt*LPMO9 action on PASC after addition of ascorbic acid 1 mM or lignin isolated from pretreated materials 10 mg/mL. Products at 5-13 min correspond to neutral sugars, 13-19 min to C1 oxidized sugars, 19-25min to C4 oxidized sugars and 25-30 min to mixed C1/C4 oxidized sugars. Peaks at 12.3, 21.8 and 31.6 min are assigned to ascorbic acid. **Figure S4.** The products of *Pc*LPMO9D action on PASC after addition of ascorbic acid 1 mM or lignin isolated from pretreated materials 10 mg/mL. Products at 5-13 min correspond to neutral sugars and 13-19 min to C1 oxidized sugars. **Figure S5.** The products of *Nc*LPMO9C action on PASC after addition of ascorbic acid 1 mM or lignin isolated from pretreated materials 10 mg/mL. Products at 5-13 min correspond to neutral sugars and 19-25 min to C4 oxidized sugars. Peaks at 12.3, 21.8 and 31.6 min are assigned to ascorbic acid. **Figure S6.** GPC chromatograms for HT-A-WS lignin before and after 24h-incubation with buffer phosphate-citrate 100 mM pH 5.0 and buffer/*Mt*LPMO9. **Table S1.** Estimated aliphatic and aromatic groups content (mmol/g) of HT-A-WS lignin before and after 24h-incubation with buffer phosphate-citrate 100 mM pH 5.0 and buffer/*Mt*LPMO9, as evaluated from ^31^P NMR. **Table S2.** Identified extractives from HT-A-WS lignin in different solvents.

## Abbreviations

LPMO: lytic polysaccharide monooxygenase
PASC: phosphoric-acid swollen cellulose
FTacV: large amplitude Fourier transform alternating current voltammetry
GPC: gel permeation chromatography
Pyr-GC/MS: pyrolysis-gas chromatography–mass spectrometry
