# Supplementary material for: Effect of lignin fractions isolated from different biomass sources on cellulose oxidation by fungal lytic polysaccharide monooxygenases
Source: Biotechnol Biofuels. 2018 Oct 28;11:296. doi: 10.1186/s13068-018-1294-6 (PMC6204277; doi:10.1186/s13068-018-1294-6)
Supplement: Supplementary file 1 — Additional file 1: Figure S1. Cyclic voltammograms of (A) HT-A-WS, (B) SE-S, (C) SE-B, (D) OS-S, (E) OS-B, (F) OS-A-S, (G) OS-A-B, (H) OS-S-LF, (I) OS-B-LF, (J) OS-A-S-LF and (K) OS-A-B-LF immobilized on a glassy carbon electrode with the use of Nafion in 100 mM tartrate buffer pH 5.0, at 50 oC between -200 and 600 mV vs Ag/AgCl for a scan rate of 5 mV/s. Figure S2. FTacV 1st to 6th Harmonic of HT-A-WS lignin immobilized on a glassy carbon electrode with the use of Nafion for a v = 50 mV/s, A = 110 mV, f = 12.3 Hz in de-aerated 100 mM tartrate buffer pH 5.0 at 50οC. Figure S3. The products of MtLPMO9 action on PASC after addition of ascorbic acid 1 mM or lignin isolated from pretreated materials 10 mg/mL. Products at 5-13 min correspond to neutral sugars, 13-19 min to C1 oxidized sugars, 19-25min to C4 oxidized sugars and 25-30 min to mixed C1/C4 oxidized sugars. Peaks at 12.3, 21.8 and 31.6 min are assigned to ascorbic acid. Figure S4. The products of PcLPMO9D action on PASC after addition of ascorbic acid 1 mM or lignin isolated from pretreated materials 10 mg/mL. Products at 5-13 min correspond to neutral sugars and 13-19 min to C1 oxidized sugars. Figure S5. The products of NcLPMO9C action on PASC after addition of ascorbic acid 1 mM or lignin isolated from pretreated materials 10 mg/mL. Products at 5-13 min correspond to neutral sugars and 19-25 min to C4 oxidized sugars. Peaks at 12.3, 21.8 and 31.6 min are assigned to ascorbic acid. Figure S6. GPC chromatograms for HT-A-WS lignin before and after 24h-incubation with buffer phosphate-citrate 100 mM pH 5.0 and buffer/MtLPMO9. Table S1. Estimated aliphatic and aromatic groups content (mmol/g) of HT-A-WS lignin before and after 24h-incubation with buffer phosphate-citrate 100 mM pH 5.0 and buffer/MtLPMO9, as evaluated from 31P NMR. Table S2. Identified extractives from HT-A-WS lignin in different solvents. [file 13068_2018_1294_MOESM1_ESM.docx]

**Effect of lignin fractions isolated from different biomass sources on cellulose oxidation by fungal Lytic Polysaccharide Monooxygenases**

Madhu Nair Muraleedharan, Dimitrios Zouraris, Antonis Karantonis,

Evangelos Topakas, Mats Sandgren, Ulrika Rova, Paul Christakopoulos, Anthi Karnaouri

**Additional Material**

*^
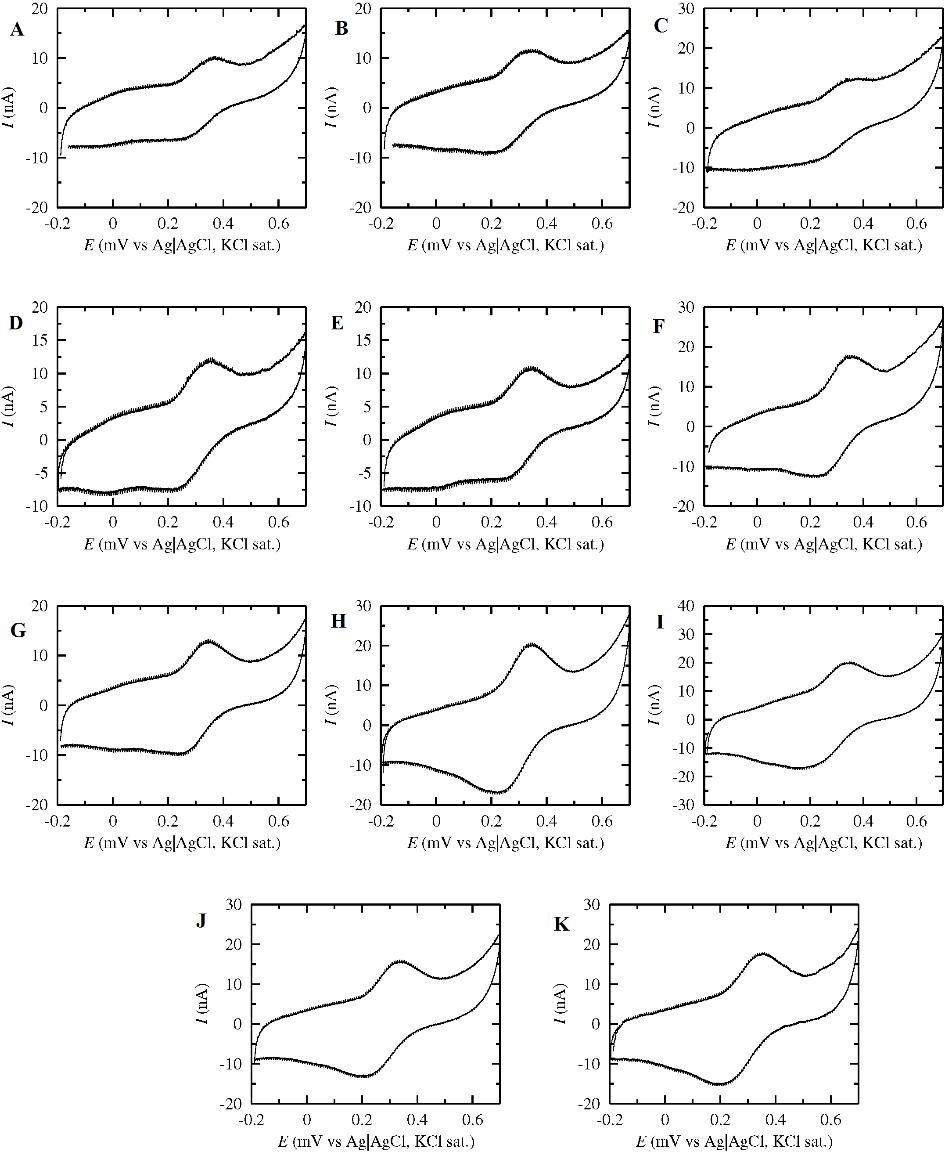
^*

**Figure S1**. Cyclic voltammograms of (**A**) HT-A-WS, (**B**) SE-S, (**C**) SE-B, (**D**) OS-S, (**E**) OS-B, (**F**) OS-A-S, (**G**) OS-A-B, (**H**) OS-S-LF, (**I**) OS-B-LF, (**J**) OS-A-S-LF and (**K**) OS-A-B-LF immobilized on a glassy carbon electrode with the use of Nafion in 100 mM tartrate buffer pH 5.0, at 50 ^o^C between -200 and 600 mV vs Ag/AgCl for a scan rate of 5 mV/s.


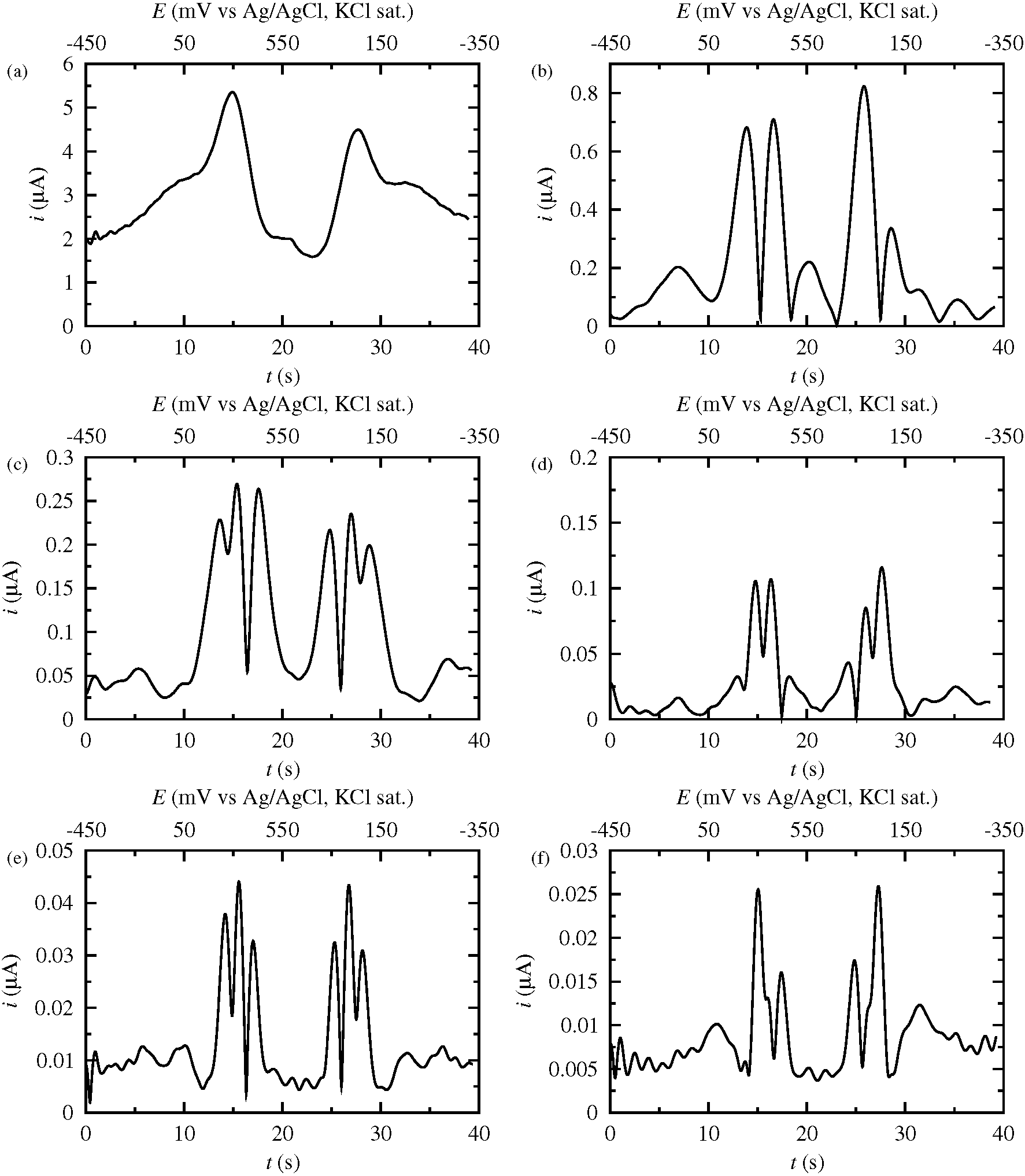


**Figure S2.** FTacV 1^st^ to 6^th^ Harmonic of HT-A-WS lignin immobilized on a glassy carbon electrode with the use of Nafion for a *v =* 50 mV/s, A = 110 mV*, f* = 12.3 Hz in de-aerated 100 mM tartrate buffer pH 5.0 at 50^ο^C.


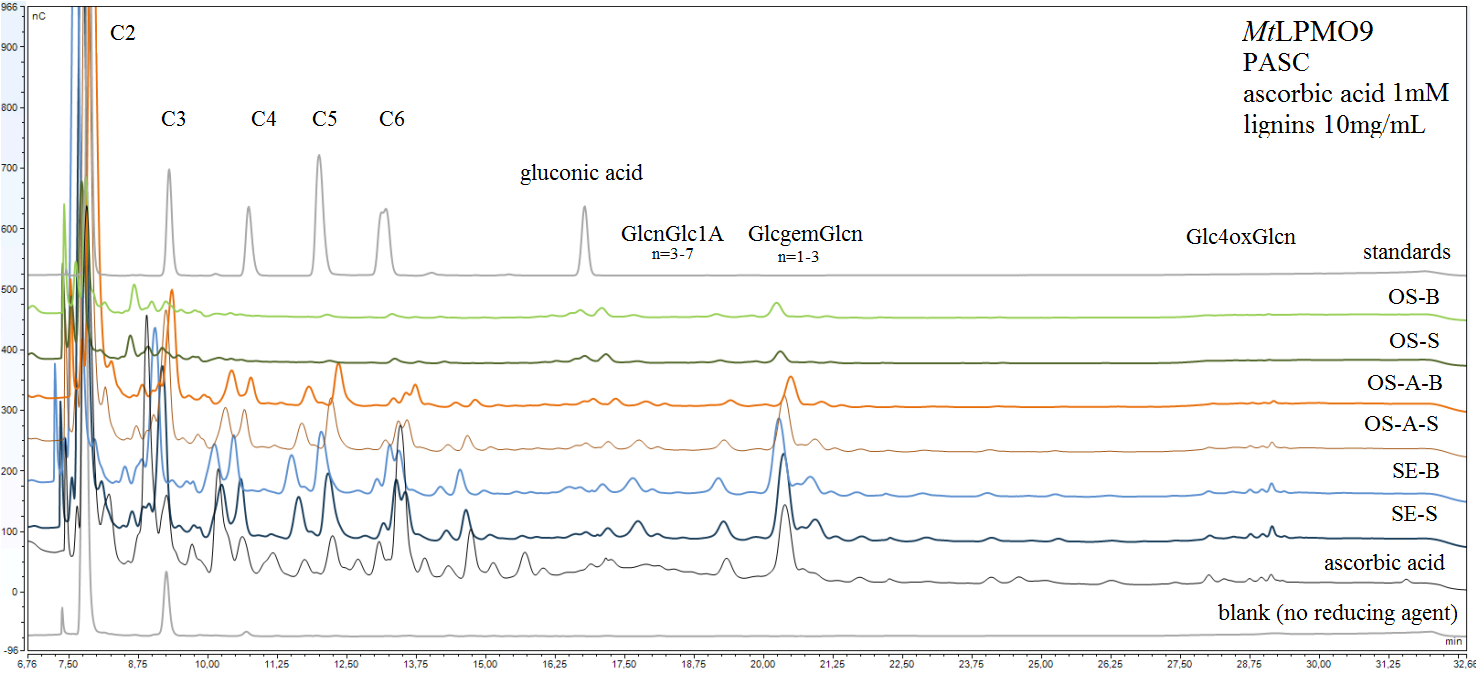


**Figure S3.** The products of *Mt*LPMO9 action on on PASC after addition of ascorbic acid 1 mM or lignin isolated from pretreated materials 10 mg/mL. Products at 5-13 min correspond to neutral sugars, 13-19 min to C1 oxidized sugars, 19-25min to C4 oxidized sugars and 25-30 min to mixed C1/C4 oxidized sugars. Peaks at 12.3, 21.8 and 31.6 min are assigned to ascorbic acid.

**
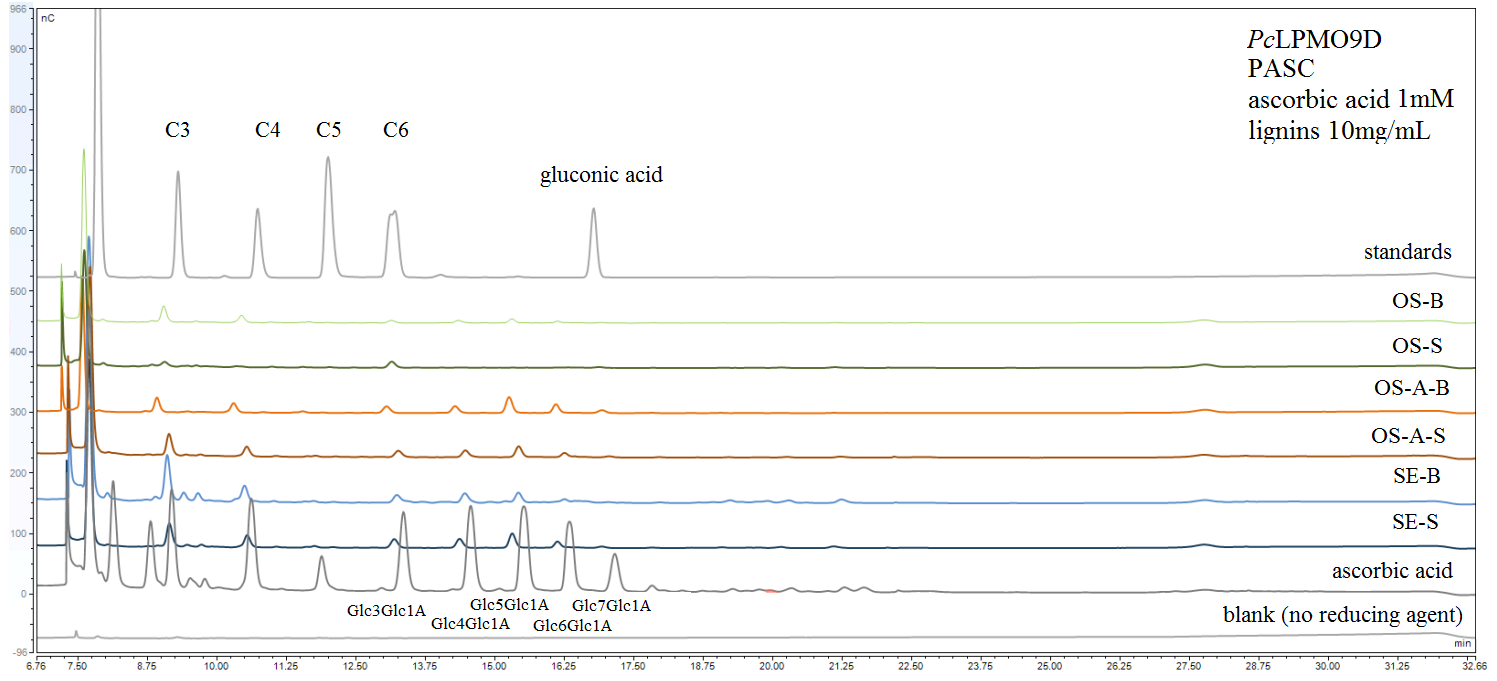
**

**Figure S4.** The products of *Pc*LPMO9D action on PASC after addition of ascorbic acid 1 mM or lignin isolated from pretreated materials 10 mg/mL. Products at 5-13 min correspond to neutral sugars and 13-19 min to C1 oxidized sugars.

**
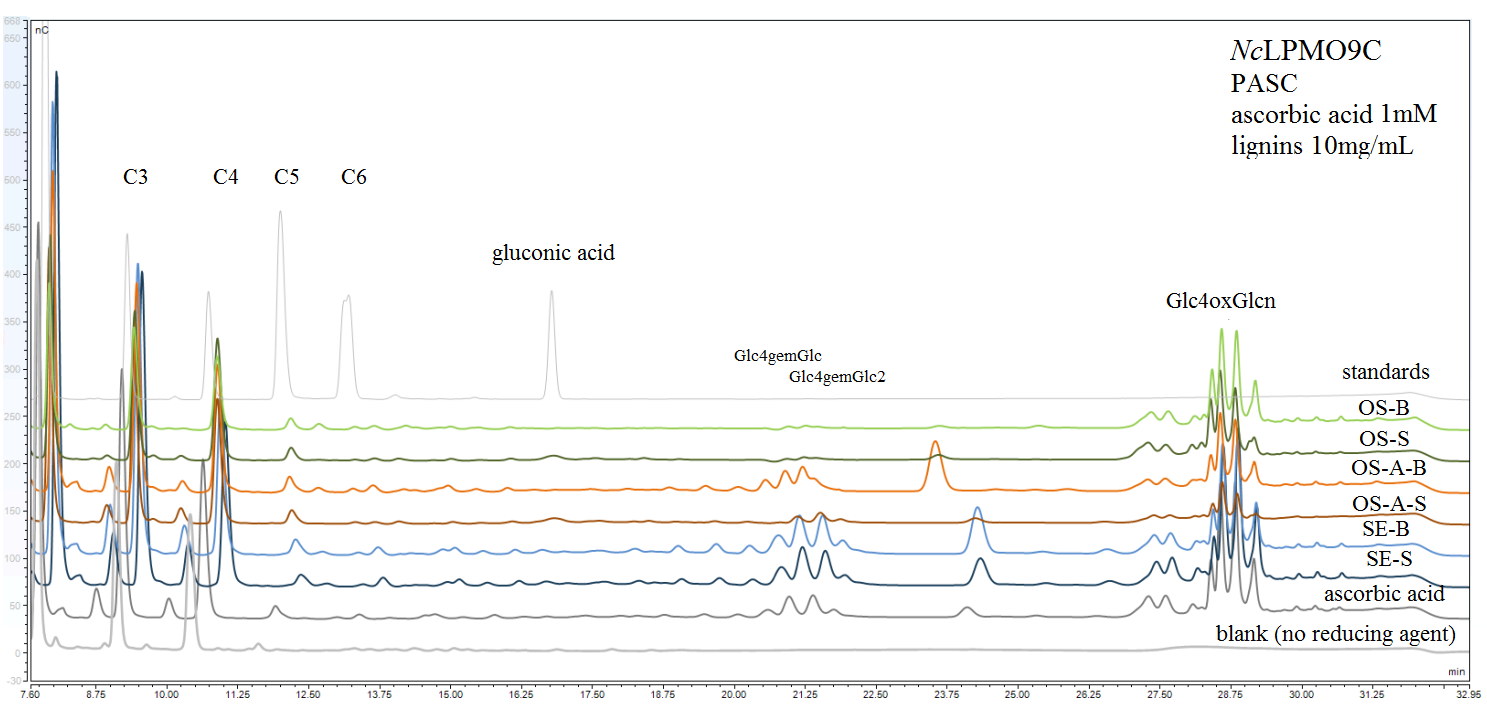
**

**Figure S5.** The products of *Nc*LPMO9C action on PASC after addition of ascorbic acid 1 mM or lignin isolated from pretreated materials 10 mg/mL. Products at 5-13 min correspond to neutral sugars and 19-25 min to C4 oxidized sugars. Peaks at 12.3, 21.8 and 31.6 min are assigned to ascorbic acid.


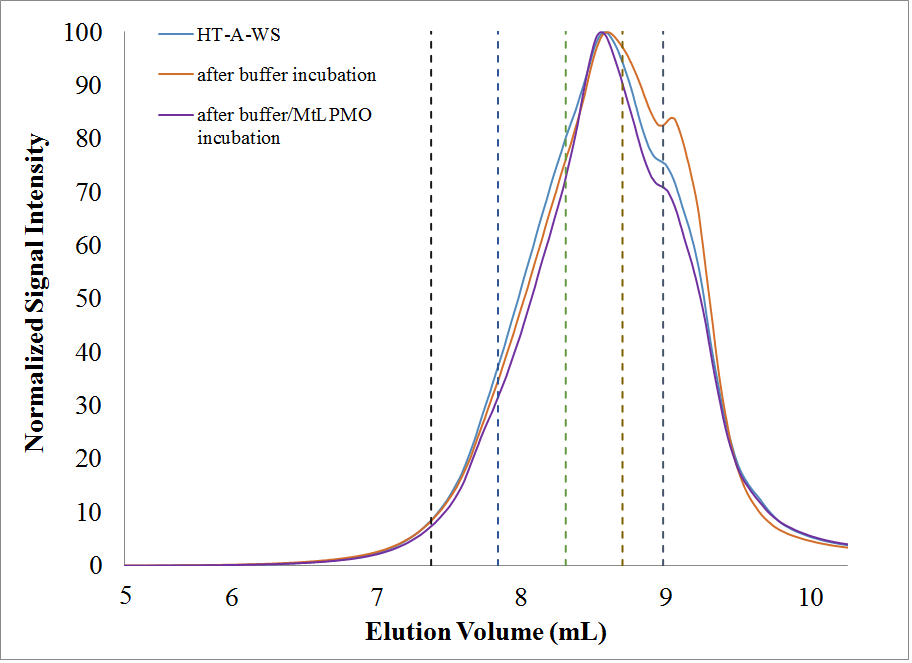


|  | ***M_n_*** | ***M_w_*** | **PDI** |
| --- | --- | --- | --- |
| *HT-A-WS* | 1262 | 3819 | 3.03 |
| *after buffer incubation* | 1198 | 3446 | 2.58 |
| *after MtLPMO9/buffer incubation* | 1291 | 2876 | 2.84 |

**Figure S6.** GPC chromatograms for HT-A-WS lignin before and after 24h-incubation with buffer phosphate-citrate 100 mM pH 5.0 and buffer/*Mt*LPMO9.

**Table S1.** Estimated aliphatic and aromatic groups content (mmol/g) of HT-A-WS lignin before and after 24h-incubation with buffer phosphate-citrate 100 mM pH 5.0 and buffer/*Mt*LPMO9, as evaluated from ^31^P NMR.

|  | **HT-A-WS** | **after incubation with buffer** | **after incubation with buffer*/Mt*LPMO9** |
| --- | --- | --- | --- |
| *aliphatic OH* | 1.08 | 1.19 | 1.17 |
| *aromatic OH* | 1.82 | 1.84 | 1.83 |
| *G/'S'* | 0.75 | 0.69 | 0.63 |
| *H/'S'* | 0.37 | 0.3 | 0.3 |
| *arom-OH/ali-OH* | 1.68 | 1.54 | 1.57 |
|  |  |  |  |
| *aliphatic* | 1.08 | 1.19 | 1.17 |
| *condensed* | 0.86 | 0.92 | 0.95 |
| *guaiacyl* | 0.64 | 0.63 | 0.59 |
| *p-OH phenol* | 0.32 | 0.27 | 0.28 |
| *total phenol* | 1.82 | 1.84 | 1.83 |
| *acidic* | 0.36 | 0.39 | 0.43 |

**Table S2.** Identified extractives from HT-A-WS lignin in different solvents.

| **identified extractive** ^a^ | **amount [%]** | | |
| --- | --- | --- | --- |
|  | **toluene** ^b^ | **ethyl acetate** ^b^ | **buffer** ^c,d^ |
| Glycerol |  | 0.5 |  |
| Vanillin | 0.8 | 1.1 |  |
| Syringaldehyde | 0.6 | 1.3 | 0.1 |
| tetradecanoic acid | 3.7 | 2.7 |  |
| *para*-coumaric acid |  | 1.2 |  |
| hexadecanoic acid | 8.0 | 10 | 0.1 |
| ferulic acid |  | 0.7 |  |
| (*Z*,*Z*)-9,12-octadecadienoic acid |  | 0.5 |  |
| oleic acid | 2.6 | 3.5 |  |
| (*E*)-11-octadecenoic acid | 0.6 | 0.5 |  |
| octadecanoic acid | 1.5 | 1.7 |  |
| eicosanoic acid |  | 0.6 |  |
| 2-monopalmitoylglycerol | 0.8 |  | 0.2 |
| 1-monopalmitoylglycerol |  | 9.0 | 0.2 |
| unidentified (m/z^max^ = 487) | 1.2 |  |  |
| 4-hydroxybutyl palmitate | 0.9 |  | 0.2 |
| unidentified (m/z^max^ = 415) | 4.9 |  |  |
| docosanoic acid |  | 1.5 |  |
| unidentified (m/z^max^ = 411) |  | 1.1 |  |
| 2-monostearoylglycerol |  |  | 0.2 |
| 1-monostearoylglycerol | 2.7 | 5.5 | 1.0 |
| tetracosanoic acid | 0.6 | 1.5 |  |
| tetracosane | 2.0 | 2.0 |  |
| hexacosanol | 1.2 | 1.1 |  |
| tetracosan-1-ol |  | 1.2 |  |
| octadecanal |  | 0.5 |  |
| hexacosane | 1.0 |  |  |
| octacosanol | 9.7 | 12 |  |
| unidentified (m/z^max^ = 481) |  | 0.8 |  |
| campesterol | 1.0 | 1.6 |  |
| stigmasterol | 1.3 | 2.0 |  |
| β-sitosterol | 3.6 | 6.2 |  |
| unidentified (m/z^max^ = 474) |  | 1.2 |  |
| unidentified (m/z^max^ = 646) |  |  |  |
| 14,16-hentriacontanedione |  | 0.7 |  |
| malic acid |  | 0.8 |  |
| lanosterol |  | 1.0 |  |

^a^: analysis was done after silylation of free hydroxyl groups with *N*,*O*-bis(trimethylsilyl)trifluoroacetamide.

^b^: extractives with a relative abundance of ≥0.5% are listed.

^c^: after acidification and extraction in ethyl acetate.

^d^: extractives with a relative abundance of ≥0.1% are listed.
